# Characterization and analytical validation of a new antigenic rapid diagnostic test for Ebola virus disease detection

**DOI:** 10.1371/journal.pntd.0007965

**Published:** 2020-01-17

**Authors:** Céline Couturier, Atsuhiko Wada, Karen Louis, Maxime Mistretta, Benoit Beitz, Moriba Povogui, Maryline Ripaux, Charlotte Mignon, Bettina Werle, Adrien Lugari, Delphine Pannetier, Sabine Godard, Anne Bocquin, Stéphane Mely, Ismaël Béavogui, Jean Hébélamou, David Leuenberger, Philippe Leissner, Takeshi Yamamoto, Patrick Lécine, Christophe Védrine, Julie Chaix

**Affiliations:** 1 BIOASTER, LYON, France; 2 FUJIFILM, Ushijima, Kaisei-machi, Ashigarakami-gun Kanagawa, Japan; 3 Centre de Recherche Et de Formation en Infectiologie de Guinée (CERFIG), République de Guinée; 4 INSERM Jean Mérieux BSL4 Laboratory, LYON, France; 5 CHRS Macenta, c/o Mission Philafricaine, Conakry, République de Guinée; University of Texas Medical Branch / Galveston National Laboratory, UNITED STATES

## Abstract

Hemorrhagic fever outbreaks are difficult to diagnose and control in part because of a lack of low-cost and easily accessible diagnostic structures in countries where etiologic agents are present. Furthermore, initial clinical symptoms are common and shared with other endemic diseases such as malaria or typhoid fever. Current molecular diagnostic methods such as polymerase chain reaction require trained personnel and laboratory infrastructure, hindering diagnostics at the point of need, particularly in outbreak settings. Therefore, rapid diagnostic tests such as lateral flow can be broadly deployed and are typically well-suited to rapidly diagnose hemorrhagic fever viruses, such as Ebola virus. Early detection and control of Ebola outbreaks require simple, easy-to-use assays that can detect very low amount of virus in blood. Here, we developed and characterized an immunoassay test based on immunochromatography coupled to silver amplification technology to detect the secreted glycoprotein of EBOV. The glycoprotein is among the first viral proteins to be detected in blood. This strategy aims at identifying infected patients early following onset of symptoms by detecting low amount of sGP protein in blood samples. The limit of detection achieved by this sGP-targeted kit is 2.2 x 10^4^ genome copies/ml in plasma as assayed in a monkey analytical cohort. Clinical performance evaluation showed a specificity of 100% and a sensitivity of 85.7% when evaluated with plasma samples from healthy controls and patients infected with Zaire Ebola virus from Macenta, Guinea. This rapid and accurate diagnostic test could therefore be used in endemic countries for early detection of infected individuals in point of care settings. Moreover, it could also support efficient clinical triage in hospitals or clinical centers and thus reducing transmission rates to prevent and better manage future severe outbreaks.

## Author summary

Ebola virus disease is a severe disease caused by Ebola virus, a member of the filovirus family, which occurs in humans and other primates. Ebola is believed to be zoonotic, however the natural reservoir is unknown. Overlapping symptoms with other endemic diseases, such as malaria and cholera, make accurate diagnostic challenging. Outbreaks of Ebola have been widespread as the consequence of the absence of available rapid, sensitive, specific, robust, and affordable licensed diagnostic test in remote areas, where outbreaks usually start. Here we have established and validated a rapid diagnostic test, which is fast, sensitive, specific, efficient, affordable, and user-friendly. Its analytical characteristics make it suitable for clinical management during Ebola virus outbreaks in remote areas. Of interest, this rapid diagnostic test detects the presence of an early viral antigen, the secreted glycoprotein, found in blood of patients shortly after infection, suggesting that it could be used to identify infected patients shortly after onset of symptoms.

## Introduction

The *Filoviridae* virus family includes 3 genera: Cuevavirus, Marburgvirus, and Ebolavirus. *Ebolavirus* (EBOV) genus is composed of six species: Zaire *ebolavirus* (ZEBOV), Soudan *ebolavirus* (SUDV), Taï Forest *ebolavirus* (TAFV), Bundibugyo *ebolavirus* (BDBV), Reston *ebolavirus* and Bombali *ebolavirus* (BOMV) [1]. Four of those species (SUDV, BDBV, TAFV and ZEBOV) infect humans and some have caused outbreaks in the past such as ZEBOV which was responsible for the recent devastating 2014 outbreak in Western Africa [2].

EBOV are enveloped single-stranded negative RNA viruses with a genome encoding seven genes. However, more than seven proteins are produced due to co-transcriptional editing and post-translational processing of the GP gene and gene products [3, 4]. The expression strategy of GP gene of all EBOV involves transcriptional editing and gives rise to different glycosylated proteins [4, 5]. As a consequence, the GP gene encodes the envelop spike glycoprotein (GP) and at least two additional non-structural secreted glycoproteins (sGP and ssGP). The sGP glycoprotein is encoded by the non-edited mRNA, representing 75% of the GP gene-specific RNA [4]. The GP protein is encoded by the addition of a single adenosine residue at the editing site, which occurs in about 20% of the GP gene-specific transcripts. In about 5% of the GP gene-specific transcripts, addition of two adenosine residues generates transcripts encoding the non-structural small secreted GP transcripts (ssGP). Furthermore, GP is endoproteolytically cleaved into surface subunit GP1 and transmembrane subunit GP2 linked by a disulfide bond to form mature GP1,2 [5]. Being the product of Ebola gene 4 co-transcriptional edition, GP1,2 and sGP have identical amino-terminal but unique carboxy-terminal sequences [6]. As a consequence of these complex transcriptional steps, the main product transcribed from the GP gene, sGP is detected in significant amount in blood from animal models and acutely infected patients [7, 8]. The multiple roles that sGP may play during infection include immunomodulatory functions such as an antigenic decoy activity, an inhibiting virus-specific neutralizing activity of GP antisera from survivors and an activity of antigen subversion of the humoral response to redirect the host immune repertoire toward epitopes that are sGP-specific or shared by the two proteins, sGP and GP [9, 10]. It has also been proposed a role for GP proteins in regulating viral production and endothelial cell barrier functions in inflammatory conditions [11, 12]. GP1,2 is also found in blood: cleavage of surface GP1,2 by the cellular metalloprotease TACE (TNFα-converting enzyme) results in GP1,2 shedding (shed GP) [7].

EBOV causes severe hemorrhagic fevers in humans, known as Ebola Virus Disease (EVD), with case fatality rates ranging from 45 to 90%. To date, the most effective way of controlling Ebola virus outbreaks is to stop human-to-human transmission [13]. Despite recent advances in EBOV vaccine development, there is still no licensed vaccine [14–16]. The greatest risk of transmission is mainly due to delayed detection and isolation of infected patients rather than from patients with already diagnosed infection [17]. Since early symptoms of EVD (fever, nausea, vomiting, diarrhea and weakness) are nonspecific and common, patients may expose family caregivers, health care workers, and other patients to the virus before the diagnosis of infection [18]. Thus, early and efficient diagnosis is a key preventive intervention that would reduce Ebola virus spreading and may ultimately control an epidemic.

Diagnosing Ebola infection is performed mainly by quantitative Reverse Transcription Polymerase Reaction Chain (RT-qPCR), in laboratory settings [8]. This cumbersome, slow and complex genetic test detects virus in blood 2 to 3 days after the onset of symptoms but requires skilled laboratory personnel and adapted technical infrastructures. Furthermore, RT-PCR requires RNA purification from blood as template and runs for 2 hours, with a total time to results around 5 hours [19]. Recently, use of the GeneXpert Ebola Assay has drastically reduced the time to results (around 2h), the requirement of technical infrastructures and qualified people, thus resulting in very sensitive diagnostic tool easy to operate in laboratory settings [20]. Despite this recent improvement, rapid diagnostic test (RDT) are urgently needed to diagnose EBOV infection in remote areas devoid of laboratory infrastructures to shortly circumvent new potential epidemics, as emphasized by the rapid spreading of recent outbreaks (2014–2016 in West Africa and May 2018 in Republic Democratic of Congo).

To develop a sensitive and rapid Ebola diagnostic test to diagnose EDV as soon as possible following onset of disease signs and clinical symptoms, we generated and screened monoclonal antibodies (mAbs) against sGP and shed GP as a surrogate of membrane GP. We then adapted silver amplification technology combined with immunochromatography methodology previously reported for influenza H5 diagnosis to generate a blood-based diagnostic test for EBOV [21]. As for influenza diagnosis, the silver amplification reaction was analyzed by an automated densitometer to determine patients’ infectious state. Using this approach, we generated a detection kit targeting sGP and to a lesser extend GP achieving a limit of detection (LOD) in plasma equivalent to 2.2 x 10^4^ genome copies/ml as assayed in a monkey analytical cohort. Clinical performance evaluation showed a specificity of 100% and a sensitivity of 85.7% with a time to test result of 15 minutes.

## Methods

### Ethics statement

All animal experimentations conducted by the INSERM Jean Mérieux BSL4 laboratory are approved by independent local and national ethical committees, and adhere to the national, European and international laws as well as provisions regarding the protection of animals for research. A4 animal facility is licensed to perform studies under Good Laboratory Practice. It has been approved for animal experimentation and breeding, by the French authorities ‘Direction départementale des services vétérinaires” (Ministry of Agriculture) D693870502, dated from June 26th, 2017 (Ministry of Agriculture). Animals are handled according to the European regulations (European Directive 2010 63/UE) and the strict procedures imposed for work in high security BSL-4 containment. Suffering of animals is avoided to the maximum possible extent.

Blood from healthy European volunteers was obtained according to procedures approved by the EFS. All donors provided informed consent to EFS. Blood from healthy African volunteers was obtained from 70 subjects (54 men and 16 women) enrolled and sampled in November 2015. The average age was 39 (min: 20; max: 67). Written informed consent was obtained from all volunteers before enrolment. This study was conducted according to the ethical standards of the Guinean Ethical committee which approved this study on 25 November 2015 under the reference 54/CNERS/15.

Samples from Ebola infected patients used in this work are frozen leftovers from clinical study performed in Macenta’s Ebola Treatment Center (ETC) during 2014 outbreak, which was approved by the Clinical Research Committee of Institut Pasteur (2015–16), the French “Commission Nationale Informatique et Liberté” (DR-2016-085; Paris, France), and the Guinean “Comité National d’Ethique pour la Recherche en Santé” (070/CNERS/15; Conakry, Guinea) [2]. As it has not been possible to obtain written informed consents from patients, an exemption is included in the Guinean “Comité National d’Ethique pour la Recherche en Santé” agreement.

### Monkey and human samples

Five cynomolgus macaques, from another study [22], were divided into two groups of 2 non-human primates and one group of 1 animal. Monkeys were infected with 10^1^ focus forming unit (FFU), 10^2^ FFU or 10^3^ FFU of ZEBOV Gabon 2002 strain, respectively. The sGP from ZEBOV Gabon 2002 strain used in this study is 98,6% identical to ZEBOV Makona (5 mutations within 364 aa, with one within the peptide leader) (S1 Fig). For most animals, blood samples were collected at day 0 (before infection), 2, 5, 7 and 8 or 9 post infection according to the animal survival. This experimentation was performed in the BSL4 animal facility of the INSERM Jean Mérieux BSL4 Laboratory.

The control groups were composed of healthy volunteers comprising 70 healthy African donors recruited by the Macenta’s Medical Center and 30 healthy European donors recruited by the “Etablissement Français du Sang” (EFS).

Samples from Ebola infected patients used in this work are frozen leftovers from a clinical study performed in Macenta’s Ebola Treatment Center (ETC) during 2014 outbreak. EBOV-infected patients were admitted on the basis of a positive RT-qPCR result with the Real-Star Filovirus Screen RT-PCR kit 1.0 (Altona Diagnostics). EBOV infection was excluded on the basis of two negative RT-qPCR tests 48 hours apart. The patients for whom EBOV infection was excluded were included in the study as febrile controls (8 samples). Patients with a confirmed diagnosis of EVD were included and classified according to outcome (21 samples). EBOV-infected patients were considered cured once their symptoms had disappeared and 2 negative EBOV RT-qPCR results had been obtained 48 hours apart (11 samples).

Each blood sample from Ebola-infected patients, febril controls and healthy African controls was drawn in BD EDTA Vacutainer tube and tested against the following disease: typhoid fever and malaria using RDTs. Blood samples from healthy African controls were also tested for HIV using RDT.

### Immunogens and recombinant proteins

ZEBOV Mayinga sGP (Ile33-Ile364, H.sapiens-tc/COD/1976/Yambuku-Mayinga, GenBank: NP_066246.1) (MednaBio, USA) and ZEBOV Makona GP (Met1-Gln650, H.sapiens-wt/GIN/2014/Kissoudougou-C15, GenBank accession number: AHX24649.2) (Sino Biological Inc., China) recombinant proteins were used as immunogens. ZEBOV Makona sGP (RBD, Met1-Phe308, H.sapiens-wt/GIN/2014/Kissoudougou-C15, GenBank accession number: AHX24649.2) (Sino Biological Inc., China) and Musoke Marburg GPdTM (MMARV GP minus the Transmembrane, Genbank Accession number: YP_001531156.1) (IBT BIOSERVICES, USA), ZEBOV Mayinga GP rGPdTM (Recombinant Ebola virus Glycoprotein minus the Transmembrane Region, Genbank Accession number: AAN37507) (IBT BIOSERVICES, USA) recombinant proteins were also used as positive and negative controls respectively for antibodies (Abs) screening. The SUDV sGP was also used in this study (Genbank Accession number ACR33190).

### Viruses

A panel of viruses ZEBOV Makona, SUDV, LASV strain AV, MARV strain Musoke and CCHFV strain 10200 was provided by the INSERM Jean Mérieux BSL4 Laboratory (Lyon, France). The MARV Popp strain was provided by the Unit of Biology of Emerging Viral Infections, Institut Pasteur, Lyon, France. Viruses were amplified using monolayer of Vero E6 cells (ATCC) incubated with each virus for 1 hour at 37°C / 5% CO_2_. Cells were then washed and cultured for several days in DMEM cell culture medium (Gibco) supplemented with 2% FCS (Eurobio Abcys). Supernatants from each viral production were collected and clarified by centrifugation at 700g during 10 minutes and finally aliquoted to generate the main stock. Below are the multiplicity of infection (MOI) and culture time for each viral production: ZEBOV Makona, MOI 0.01, cultured for 8 days; SUDV, MOI 0.001, cultured for 7 days; LASV strain AV, MOI 0.01, cultured for 3 days; MARV strain Musoke, MOI of 0.001, cultured for 4 days; MARV strain Popp, MOI of 0.1, cultured for 4 days; CCHFV strain 10200, MOI 0.0001, cultured for 2 days.

### Viral titration

EBOV virus titration from either viral supernatants or from infected plasmas was performed with Vero E6 cells plated in 12-wells microplates 24h prior to infection. Infectious supernatants or infected plasmas were tenfold serially diluted in DMEM cell culture medium (Gibco) supplemented with 2% FCS (Eurobio Abcys). Vero E6 cells were infected with the serial dilutions and incubated for 1 hour at 37°C / 5% CO_2_. Semi solid medium containing 1.6% of carboxymethyl cellulose (CMC) in DMEM with 2% FCS was then added (vol / vol). Plates were incubated during 7 days at 37°C / 5% CO_2_. Infectious foci were detected by incubation with a GP EBOV specific monoclonal antibody produced in mice (generously provided by Laurent Bellanger and Fabrice Gallais, LI2D Laboratory, CEA, Marcoule, France), followed by incubations with an Alkaline phosphatase-conjugated polyclonal anti-mouse IgG (Sigma Aldrich) and a 1-step NBT/BCIP plus Suppressor (Thermo Fisher Scientific). For each sample, results are expressed in Focus Forming Units (FFU) per ml of blood. All experiments performed on infectious material were performed in the INSERM Jean Mérieux BSL4 Laboratory.

### RT-qPCR and genome copies quantification

Before RNA extraction performed outside the BSL4 Laboratory, infectious samples were inactivated by addition of AVL buffer and absolute ethanol according to manufacturer instructions (QIAGEN) [23]. Total RNA was then extracted using the QIAamp Viral RNA kit (QIAGEN) following the manufacturer specifications. EBOV NP gene was detected using one step RT-qPCR previously described by Huang et al. and absolute EBOV genome quantification was determined with reference to the standard curve obtained from serial dilutions of a standardized plasmid including a part of the NP gene (developed by the French National Reference Center for Viral Hemorrhagic Fevers) [24]. For each sample, PCR reaction was performed in duplicate on a CFX 96 Touch system (BIORAD).

### Monoclonal antibodies (mAbs) generation

5 Balb/c mice (8–12 weeks of age) per immunogen were immunized by intraperitoneal or foot pad injections using either sGP or GP proteins (total of 20 mice: 5 mice x 2 injection sites x 2 antigens) emulsified in Freund's complete adjuvant (Sigma) (first injection) or incomplete adjuvant (Sigma) (boost injections) (5–10 μg of proteins every 2 weeks). Three days after the last injection, spleen cells (intraperitoneal injections) or popliteal lymph node cells (foot pad injections) were fused with SP2/0 myeloma cells (Sp2/0-Ag14 (ATCC CRL-1581), using polyethylene glycol (PEG 1500) (Sigma). Hybridomas were grown in DMEM culture medium (Sigma) containing 20% Fetal Calf Serum (FCS) (Eurobio, France), penicillin (100 IU/ml) and streptomycin (100 μg/ml) and supplemented with hypoxantine (1×10^−4^ M), aminopterin (4×10^−7^ M) and thymidine (1.6×10^−5^ M) (HAT) (Sigma). After ten to fourteen days of culture, secreting hybrids were identified by analysis of culture supernatants by indirect enzyme-linked immunosorbent assay (ELISA). Hybridomas from selected antibody were cloned by limiting dilution and processed according to conventional methods. Clones secreting antibody of desired reactivity were expanded in 25 and 75 cm^2^ flasks (Nunc, Denmark), harvested and cryopreserved in 40% FCS, 10% Dimethylsulfoxide (DMSO) (Sigma) and 50% RPMI-1640 culture medium (Sigma).

### ELISA for monoclonal antibodies (mAbs) selection and specificity

Briefly, Nunc Maxisorp ELISA plates were coated with the immunogens, recombinant sGP or GP protein, blocked with PBS + 1% BSA and incubated with sera or cell culture supernatant. After a washing step, appropriate dilution of HRP-conjugated sheep anti-mouse Ig was added to the wells and incubated for 1 hour at room temperature (RT) following by a washing step. The reaction was revealed with TMB one component substrate (RD-Biotech). The reaction was stopped with 20% H_2_SO_4_ and the optical density (OD) measured by a Multiscan EX ELISA reader (ThermoFischer) at 450 nm & 620 nm. To evaluate the specificity of the generated mAbs, ELISA were also using GP and sGP from different EBOV viruses.

### Identification of pair of mAbs recognizing Ebola sGP or GP

An anti-mouse immunoglobulin Ab and mAbs EE8, XC2, WE7, DB4, VB5, FE5 or FD3 were immobilized onto nitrocellulose membranes (Millipore, USA) to generate control and test lines, respectively. A reagent pad containing WE7, DB4, VB5, EE8, FE5, FD3 or XC2 mAbs conjugated with colloidal gold (British Biocell International, UK) was placed between the sample application point and test line. Positivity was determined by visual inspection. Screening strategy to select mAbs specific for sGP, GP or both is depicted in S2 Fig.

### Development of the Ebola sGP Detection Kit with silver amplification immunochromatography technology

An anti-mouse immunoglobulin and EE8 Ab were immobilized onto nitrocellulose membranes (Millipore, USA) to generate the control and test lines, respectively. A reagent pad containing XC2 mAb conjugated with colloidal gold (British Biocell International, UK) was placed between the sample application point and test line. The test strip was then placed in a FUJI DRI-CHEM IMMUNO AG cartridge (FUJIFILM, Japan). Results express as delta OD values were calculated by FUJI DRI-CHEM IMMUNO AG1 analyzer (FUJIFILM, Japan) based on OD values before and after silver amplification reaction.

To determine the LOD of test line, delta OD values of test conditions without antigen were analyzed. The passive adsorption of gold label detection antibody gave a background signal of 18 delta OD, thus constituting the cut-off of the test line. We also evaluated experimentally the cut-off of the sGP Detection kit with plasma samples from healthy donors from Europe (30) and Guinea (70). Mean of delta OD was 3.66 (standard deviation of 3.74) and 5.2 (standard deviation of 3.9) with samples from Europe or Guinea respectively. The cut-off value taken as mean + 3 SD is 13.56 and 16.9 when using samples from Europe or Guinea respectively, which is in agreement with the cut-off used of delta OD 18 applied in this study.

### ASSAY optimization steps

The lateral flow assay optimization includes several steps. First, the spraying concentration of the capture antibody on the nitrocellulose membrane was determined to maximize capture efficiency of the antigen. Second, the gold nanoparticle conjugate was improved. F(ab’)2 digestion protocol and the concentration of gold nanoparticle at the conjugation step were finely tuned. The optimal conjugate concentration was set to maximize the sensitivity of the assay. Finally, the sample dilution buffer was supplemented with human anti-murine antibodies (HAMA) blocker to improve assay specificity.

### Specificity evaluation with plasma from healthy African donors and healthy European donors

Plasma from African and European healthy donors were 5-fold diluted with an extraction buffer (Tris EDTA buffer pH7.7 containing 0.1% Casein (Wako, Japan), 1% BIGCHAP (Dojindo, Japan)). 5-fold diluted plasma (140 μl final volume) was applied to the sample application point of the diagnostic cartridge. Then, the cartridge was inserted into FUJI DRI-CHEM IMMUNO AG1 analyzer (FUJIFILM, Japan), in which silver amplification was performed automatically. The results were interpreted based on the displayed information and the measured delta OD.

### Repeatability evaluation

Repeatability was evaluated by analyzing the same sample 10 times with the EBOV sGP Detection Kit following by measurement of the delta OD by the FUJI DRI-CHEM IMMUNO AG1 analyzer (FUJIFILM, Japan). Two dilutions of a culture supernatant from Makona Ebola infected cells (3.00 x 10^6^ FFU/ml) were used: 10-fold and 250-fold dilutions in naive human plasma. Means and standard deviations were then calculated.

### Analytical sensitivity evaluation

ZEBOV Makona virus supernatant (3 x 10^6^ FFU/ml) was 5-fold serial diluted (from 1/5 to 1/15 625) into naive human plasma provided by “Etablissement Français du Sang” (Lyon, France). 140 μl of each dilution were loaded in the cartridge. In parallel, 140 μl of each dilution were extracted for EBOV NP gene detection as described above. The limit of detection as viral genome copy number was assigned to the first positive result above the cut-off value (delta OD 18) obtained with the FUJI DRI-CHEM IMMUNO AG1 analyzer.

Analytical sensitivity evaluation was also performed with plasma from Ebola virus infected monkey. Plasma collected 7 days after infection from one monkey infected with 10^2^ FFU was 5-fold serial diluted (from 1/5 to 1/15 625) in naive human plasma or whole blood. 70 μl of each dilution in whole blood were absorbed on a blood pick apparatus (which contains an absorbent sponge). The sponge is released in the extraction buffer and mixed vigorously 30–40 times. Plasma was extracted from the whole blood with the help of a Blood cell separation cylinder (which contains a separation membrane). This plasma extraction system is manufactured by FUJIFILM. 140 μl of this extracted plasma were loaded in the cartridge and read with the AG1 analyzer. Dilution in naive human plasma followed the same process and 140 μl of the mix plasma/buffer was loaded in the cartridge and analyzed. In parallel, RT-qPCR was performed only on the plasma dilutions to evaluate the genome copy number of each dilution. For each sample, genome copy numbers were compared to the AG1 analyzer values to determine the LOD.

### Viral cross-reactivity evaluation

The specificity of EBOV sGP Detection Kit was performed against other viruses responsible of viral hemorrhagic fever (VHF) outbreaks in Africa. For technical reasons, each infectious supernatant [SUDV (6.16 x 10^6^ FFU/ml), MARV Musoke (9.17 x 10^6^ FFU/ml) and Popp (7.90 x 10^6^ FFU/ml) strains, LASV AV strain (5.80 x 10^5^ FFU/ml), and CCHF 10200 strain (3.70 x 10^6^ FFU/ml)] was pre-diluted 10-fold with naive human plasma from the "Etablissement Français du Sang". Samples were then diluted 5-fold with the extraction buffer and 140 μL from the final dilution (1/50) was applied to the cartridge and then read by the FUJI DRI-CHEM IMMUNO AG1 analyzer (FUJIFILM, Japan). Positive control solutions were obtained by diluting ZEBOV sGP recombinant protein in naive human plasma and then 1/5 in assay buffer to give final concentrations of 125 ng/ml and 31.3 ng/ml. Negative control consisted in a 5-fold dilution of a naive human plasma with assay buffer. The experiment was done in triplicate and the results were presented with a positive or negative detection of these VHF viruses.

### Clinical sensitivity evaluation with plasma from Ebola infected patients

The clinical sensitivity of EBOV sGP Detection Kit was evaluated on 40 human plasmas collected during West Africa Ebola outbreak (Macenta—Guinea) [25]. The positive samples were previously selected based on a RT-qPCR positive value (Ct value<34 cycles). Plasma samples were diluted 5-fold with the assay buffer and 140 μL were analyzed by the FUJI DRI-CHEM IMMUNO AG1 analyzer (FUJIFILM, Japan) [25]. The negative control was a dilution (1/5) of a naive human plasma with assay buffer and positive controls were as described above. The results were expressed with delta OD values.

## Results

Based on sequential screening by EIA, ELISA and immunochromatography (see methods and S2 Fig) we selected a pair of mAbs: EE8 and XC2 as capture and detection mAbs respectively, since they were giving the lowest LOD in LFA. They were subsequently used in an Ebola sGP Detection Kit, a diagnostic test relying on silver amplification coupled to immunochromatography. Both EE8 and XC2 mAbs recognize sGP and GP from Mayinga and Makona, even though we note a better recognition for the secreted proteins as depicted in the Table 1.

**Table 1:** Evaluation of antibody detection of ZEBOV Mayinga and Makona variants of sGP or GP, and Marburg GP.

Ag used to immunize mice	Clone name	Indirect immunoassay Coated Ag					Antibody specificity
		ZEBOV Mayinga GP	ZEBOV Makona GP	ZEBOV Mayinga sGP	ZEBOV Makona sGP	Musoke Marburg GPdTM	
**ZEBOV Mayinga sGP**	DB4	**++++**	**++++**	**++++**	**++++**	**-**	**ZEBOV GP & sGP**
**ZEBOV Mayinga sGP**	RB6	**++++**	**++++**	**++++**	**++++**	**-**	
**ZEBOV Mayinga sGP**	SE2	**++++**	**++++**	**++++**	**++++**	**-**	
**ZEBOV Mayinga sGP**	VB5	**++++**	**++++**	**++++**	**++++**	**-**	
**ZEBOV Mayinga sGP**	WE7	**+++**	**+++**	**++++**	**++++**	**-**	
**ZEBOV Mayinga sGP**	WF2	**++++**	**+++**	**++++**	**++++**	**-**	
**ZEBOV Mayinga sGP**	XC2	**+++**	**++**	**++++**	**++++**	**-**	
**ZEBOV Mayinga sGP**	XE5	**++++**	**++++**	**++++**	**++++**	**-**	
**ZEBOV Makona GP**	EE8	**++**	**+++**	**++++**	**++++**	**-**	
**ZEBOV Makona GP**	FD3	**++++**	**++++**	**++++**	**++++**	**-**	
**ZEBOV Makona GP**	FE5	**++++**	**++++**	**+++**	**++++**	**-**	
**ZEBOV Makona GP**	JA7	**++++**	**++++**	**++++**	**++++**	**-**	
**ZEBOV Makona GP**	JC1	**++++**	**++++**	**++++**	**++++**	**-**	
**ZEBOV Makona GP**	GC1	**+**	**+**	**++++**	**++++**	**-**	**ZEBOV sGP**
**ZEBOV Makona GP**	IF5	**-**	**-**	**+**	**+++**	**-**	
**ZEBOV Makona GP**	JC2	**++++**	**++++**	**-**	**-**	**-**	**ZEBOV GP**
**ZEBOV Makona GP**	KE4	**++++**	**++++**	**-**	**-**	**-**	
**Optical density**	**< 0.5**	**0.5–1**	**1–1.5**	**1.5–2**	**> 2**		
**Intensity scale**	**-**	**+**	**++**	**+++**	**++++**		

### Evaluation of Ebola sGP detection Kit LOD, repeatability, cross-reactivity and stability

**Fig 1:**
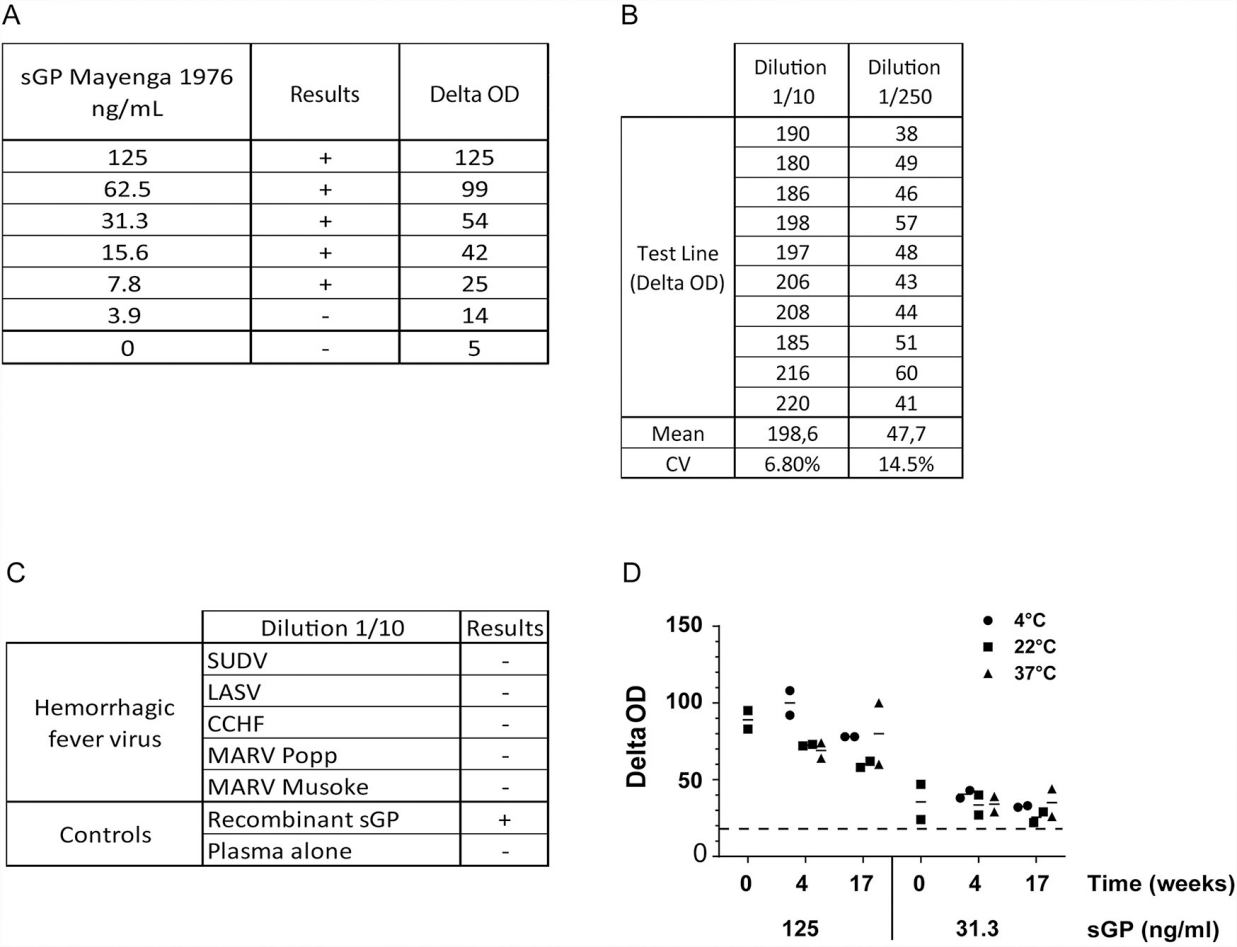
Evaluation of Ebola sGP Detection Kit: LOD of recombinant sGP, repeatability, cross-reactivity and stability. (A) Recombinant ZEBOV Makona sGP was diluted in human plasma at the indicated concentration and subjected to Ebola sGP detection kit test. (B) Supernatant of infected Vero cells (3 x 10^6^ FFU/ml) was diluted 10 and 250 times (1.03 x 10^8^ and 2.11 x 10^6^ RNA copies/ml respectively) in human plasma and subjected to sGP Detection Kit. Results are test line delta OD of single measurement (n = 10), mean and coefficient of variation (%) as indicated. (C) Supernatant of Vero cells infected with several different viruses responsible for hemorrhagic fevers were diluted 1/10 in human plasma and subjected to sGP Detection Kit. Results are test line delta OD of single measurement. (D) Kit long term stability was assayed at week 0, 4 and 17 on plasma spiked with 125 ng/ml or 31.3 ng/ml of recombinant ZEBOV Makona sGP protein as indicated. sGP Detection Kit was stored at 4°C (white bars), 20°C (grey bars) or 37°C (black bars). Results are test line delta OD of measurement of duplicate and mean indicated as an horizontal bar for each time points and storage conditions. Only one value is indicated at week 0, as tests were freshly manufactured.

We first established the Ebola sGP Detection Kit LOD by serial dilutions of recombinant sGP protein in human plasma (Fig 1A). The minimal concentration of spiked sGP detected is 7.8 ng/ml (based on a cut off value of delta OD 18), corresponding to 60 pM of protein, representing a low LOD, in agreement with previously reported LOD of silver-enhanced immunochromatography [26]. As we only use 140 μl of sample per test, our diagnostic kit detects 1.09 ng of spiked sGP (Fig 1A).

Next, we analyzed Ebola sGP Detection Kit test repeatability on viral supernatant from Vero-infected cells, diluted 10 (1.03 x 10^8^ RNA copies/ml) and 250 times (2.11 x 10^6^ RNA copies/ml) in human serum. The coefficient of variation (CV) was better for dilution 1/10 (CV = 6.9%) than for dilution 1/250 (CV = 14.4%) (Fig 1B). Even though there is a slight variation in CV values, all of the 10 supernatant replicates for both dilutions gave positive results, showing the robustness of Ebola sGP Detection Kit test (Fig 1B).

We then assayed Ebola sGP Detection Kit test cross-reactivity against other hemorrhagic fever viruses. Culture supernatants from cells infected with SUDV, Lassa fever virus (LASV), Crimean-Congo hemorrhagic fever (CCHF) virus as well as Marburg virus (MARV) strains Musoke and Popp were used. Results of Fig 1C showed that Ebola sGP Detection Kit does not display any cross-reactivity with other virus causing hemorrhagic fevers while recognizing recombinant sGP. However, SUDV strain could not be detected.

Finally, we performed a real time shelf storage stability test of the Ebola sGP Detection Kit. Human plasma samples were spiked with 125 or 31.3 ng/ml of ZEBOV Makona sGP recombinant protein, used as standard solutions and were preserved at -80°C. Ebola sGP Detection Kits were kept either at 4°C, 20°C or 37°C. Ebola sGP Detection Kit tests were performed in duplicates at day 0 or after four or seventeen weeks with both standard solutions. All samples gave positive results independently of the week and the storage temperature considered (Fig 1D). Furthermore, even with the lowest concentration of spiked sGP and storage at 37°C for seventeen weeks, Ebola sGP Detection Kit test retrieves delta OD values very close to day 0 (35.5 delta OD versus 35 delta OD respectively for the 31.3 ng/ml solution), demonstrating very good stability over time at different storage temperatures (Fig 1D).

### Evaluation of Ebola sGP detection kit LOD with culture supernatants and infected monkey plasmas

**Fig 2:**
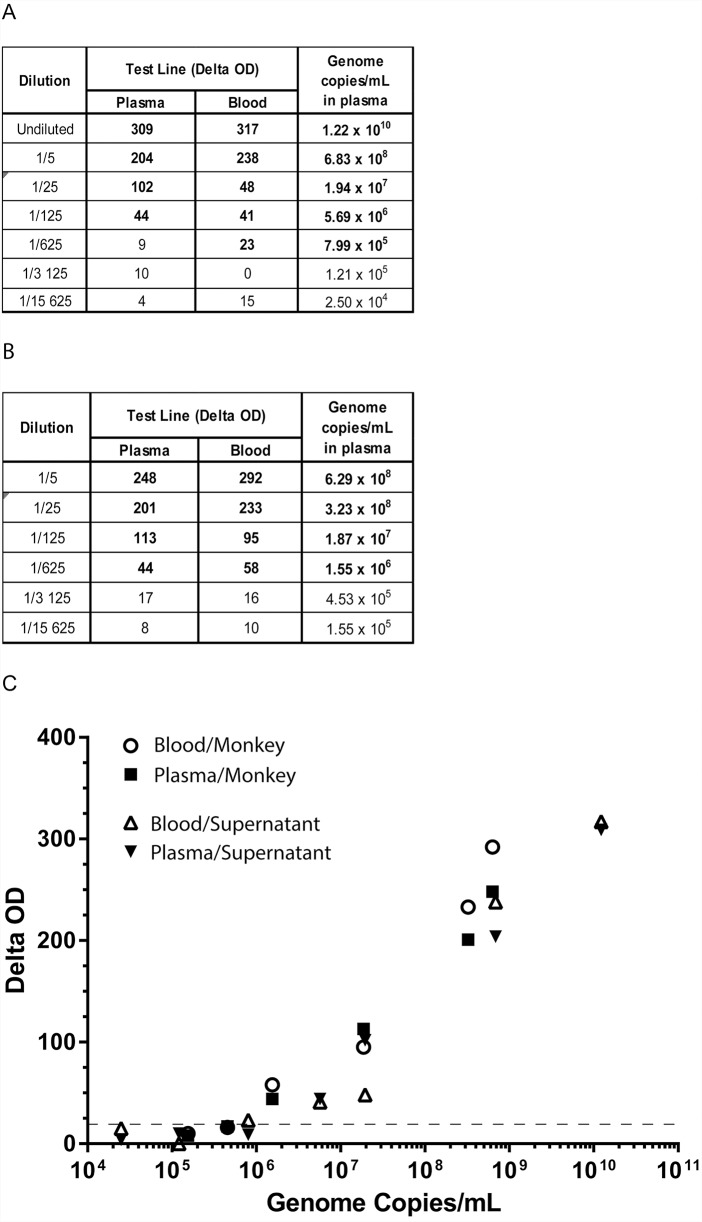
Evaluation of Ebola sGP Detection Kit LOD with supernatant from cell culture and infected monkey plasma. (A) Supernatant of Vero cells containing ZEBOV viral particles at 3 x 10^6^ FFU/ml was diluted as indicated in human plasma or blood and subjected to RT-qPCR and Ebola sGP Detection Kit. Results are test line delta OD of single measurement or mean of genome copies/ml from RT-qPCR duplicates performed on dilutions made in plasma. Sample positively detected by sGP Detection Kit are in bold characters. (B) Monkey infected intravenously with 10^2^ FFU of EBOV Gabon 2002 was bled at day 7 post-infection. The plasma containing 2.15 x 10^10^ genome copies/ml was subsequently diluted in human plasma or blood. Genome copies and sGP Detection Kit test were then performed. Results are test line delta OD (positive Ebola sGP Detection Kit tests are in bold characters) and mean of genome copies/ml from RT-qPCR duplicates performed on dilutions made in plasma. (C) Delta OD and related Log 2 genome copies/ml of samples tested in Fig 2A and 2B diluted human plasma (black) or in human blood (white) were plotted, adjusted R^2^ values calculated with positive values, are 0.95 and 0.925 for monkey plasma and cell supernatant respectively. Linear regression analysis showed no effect of human matrices on Delta OD measurement (Student p values of 0.797 and 0.37 for monkey plasma and cell supernatant respectively). Culture supernatant diluted in human blood and plasma are shown as white circle and black square respectively. Infected monkey plasma diluted in human blood and plasma are shown as white and black triangle respectively.

We next evaluated the LOD of our detection kit with ZEBOV Makona particles from cell supernatant undiluted or serially diluted in human plasma or blood. Genome copy numbers were measured in parallel for each dilution in plasma. The Ebola sGP Detection Kit gave positive results (i.e. test line delta OD value above 18, bold characters) for supernatant dilutions in both human plasma and blood (Fig 2A). These dilutions have genome copies concentrations ranging from 1.22 x 10^10^ to 2.5 x 10^4^ genome copies/ml. Thus, in those conditions Ebola sGP Detection Kit has limits of detection equivalent to 5.69 x 10^6^ and 7.99 x 10^5^ genome copies/ml when dilutions are made with plasma and blood respectively.

As ZEBOV produced by Vero cells may not reflect production following infection of a whole organism, i.e. ratio of viral proteins may differ, especially sGP, we next evaluated the effect of human matrix (blood or plasma) on Ebola sGP Detection Kit LOD to detect ZEBOV in plasma of an infected monkey. To this end, we analyzed genome copy number and Ebola sGP Detection Kit test on monkey plasma at 7 days post-infection (inoculated with 10^2^ FFU of ZEBOV Gabon). Monkey plasma was serially diluted in human plasma or blood. Ebola sGP Detection Kit gave positive results for conditions where genome copy numbers were as low as 1.55 x 10^6^ copies/ml when human plasma and blood were used as matrix (Fig 2B). Of note, linear regression analysis of Delta OD and genome copy log values showed that Ebola sGP Detection Kit test gave similar results independently of the human matrix used to dilute infected monkey plasma or cell supernatant (Student p values > 0.05, Fig 2C).

### Evaluation of Ebola sGP detection kit analytical sensitivity in infected monkey cohort

To sharpen analytical sensitivity, we then analyzed plasma samples from monkeys infected with different doses of ZEBOV Gabon (10^1^, 10^2^ or 10^3^ FFU) and collected at different times post-infection. Results displayed on Table 2 showed that the Ebola sGP Detection Kit gave positive results when monkey plasmas contain around 2 x 10^4^ genome copies/ml corresponding to viral titers of 5 x 10^2^ FFU/ml. Moreover, all tests showed positivity for plasmas from monkeys infected with all three doses of ZEBOV from day 5 to day 9, including very high viral load samples, up to 10^9^ or 10^10^ RNA copies/ml. Since in human blood, the highest viral load is lower than 10^10^ RNA copies/ml, we assume the test may not be susceptible to Hook effect, but further experiments should validate this hypothesis [27]. However, the genome copies detection for monkey 1 infected with 10^1^ FFU at day 5 was positive for only one RT-qPCR replicate (noted with *). This was also the case for genome copy analysis at day 2 for monkey 1 infected with 10^2^ FFU and monkey 1 infected with 10^3^ FFU (with 3.49 X 10^4^ and 1.26 x 10^4^ genome copies/ml respectively) with again only one positive RT-qPCR replicate. Thus, the limit of RT-qPCR detection is above 3.49 x 10^4^ genome copies/ml in settings used in our BSL4 laboratory. By comparison Ebola sGP Detection Kit test was positive whenever genome copies gave robust positive results (i.e. both RT-qPCR replicates were positive) as well as for one monkey infected with 10^1^ FFU at day 5 post-infection (biological replicate 1, one positive RT-qPCR replicate at 2.21 X 10^4^ copies/ml). Therefore, the relative detection limit of Ebola sGP Detection Kit was 2.21 x 10^4^ genome copies/ml. This indicated an analytical detection sensitivity close to the order of magnitude obtained by RT-qPCR, at least in our settings. We also note a false positive result with the biological replicate 2 infected with 10^1^ PFU at day 0. Over all the negative samples tested from human or monkey origin during this work (see below), this was the only false positive result.

**Table 2:** Evaluation of Ebola sGP detection kit analytical sensitivity in infected monkey cohort.

		Biological replicate 1			Biological replicate 2		
Infection dose	Day post-infection	Test line (Delta OD)	Concentration (RNA Copy/mL)	Titer (FFU/mL)	Test line (Delta OD)	Concentration (RNA Copy/mL)	Titer (FFU/mL)
10^1^	0	18	ND	ND	**20**	**ND**	**ND**
	2	17	ND	ND	0	ND	ND
	**5**	**229**	**2.21 x 10**^4^*	**5.20x 10**^**2**^	**45**	**5.85 x 10**^**5**^	**1.00 x 10**^**2**^
	**7**	**283**	**1.14 x 10**^**9**^	**1.05x 10**^**6**^	**304**	**5.35 x 10**^**6**^	**3.27 x 104**
	**9**	**246**	**1.87 x 108**	**5.35x 10**^**4**^	**271**	**4.60 x 10**^**5**^	**1.50x 10**^**2**^
10^2^	0	6	ND	ND	10	ND	ND
	2	8	**3.49 x 10**^4^*	ND	4	ND	ND
	**5**	**301**	**8.00 x 10**^**7**^	**8.20x 10**^**4**^	**286**	**8.58 x 10**^**8**^	**1.00 x 10**^**5**^
	**7**	**252**	**3.66 x 10**^**9**^	**1.40x 10**^**6**^	**262**	**2.15 x 10**^**10**^	**2.02 x 10**^**6**^
	**9**	**248**	**5.71 x 10**^**8**^	**2.30x 10**^**4**^	**177**	**7.17 x 109**	**1.00x 10**^**6**^
10^3^	0	NT	NT	NT	2	ND	ND
	2	3	**1.26 x 10**^4^*	ND	9	ND	ND
	**5**	**311**	**2.59 x 10**^**8**^	**1.16 x 10**^**5**^	**93**	**4.43 x 10**^**6**^	**1.00 x 10**^**1**^
	**7**	**222**	**1.02 x 10**^**9**^	**5.92 x 10**^**5**^	**280**	**1.47 x 10**^**6**^	**1.25 x 10**^**5**^
	**9**	**273**	**2.36 x 10**^**8**^	**5.97x 10**^**4**^	**243**	**1.75 x 10**^**5**^	**1.50x 10**^**3**^

Note: Infection dose (FFU/biological replicates); Test line (Ebola sGP Detection Kit test line delta OD positive values in bold characters); Concentration (mean of duplicates genome copies/ml), values noted *: samples with only one replicate positive out of two; Viral titer (FFU/ml); ND: not detected, NT: not tested.

### Ebola sGP detection kit specificity evaluation

**Fig 3:**
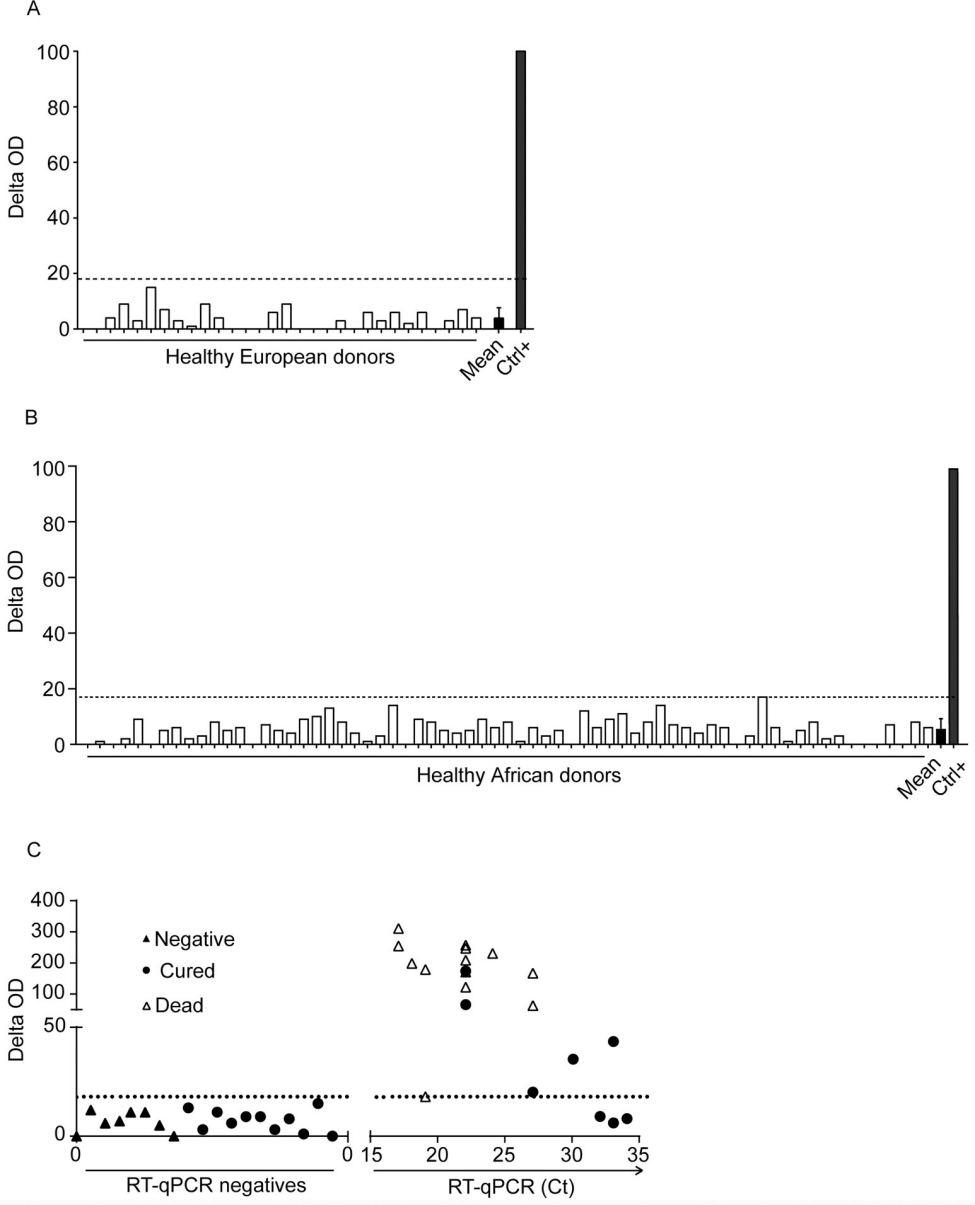
Clinical specificity and sensitivity evaluation. Plasmas from Healthy European (n = 30) (A) and African (n = 67) (B) donors were subjected to Ebola sGP Detection Kit test. Results are express in delta OD for each donor (white bars), donors mean + S.D. (black bars) and plasma spiked with 125μg/ml of sGP (Ct+ grey bar). (C) Plasmas from EBOV-infected patients were subjected to RT-qPCR and sGP Detection Kit. Results are test line delta OD of single measurement or mean Ct values of qRT-PCR duplicates (performed within the ETC of Macenta). EBOV patients were stratified based on disease outcome (Death: white triangle, Cured: black round) and plotted together with EBOV negatives patients (Negative: black triangle).

We first aimed to optimize test conditions in order to achieve the lowest background when using negative human European healthy plasma samples. Following optimization of test conditions, we obtained an average test line delta OD value of 3.66 with standard deviation of 3.74 (Fig 3A). Those same conditions were then used on a cohort of healthy Africans donors to analyze Ebola sGP Detection Kit specificity. All the 70 plasmas of African healthy donors were negative, thus the specificity of Ebola sGP Detection Kit was 100% (Fig 3B). Of note, in this assay, the delta O.D average value was 5.2 with a standard deviation of 3.9 (Fig 3B).

### Clinical sensitivity evaluation with plasma from Ebola infected patients

Finally, we assessed Ebola sGP Detection Kit clinical sensitivity and specificity with plasma samples from infected individuals [25]. Patients were included in the cohort based on RT-qPCR test positivity, i.e. when Ct values were inferior to 34 cycles [25]. Out of 21 patients diagnosed by RT-qPCR, 18 patients were positive with the Ebola sGP detection Kit test, giving a sensitivity of 85.7% (Fig 3C). Out of the 3 patients not detected by Ebola sGP Detection Kit, all are close to the RT-qPCR detection limits with Ct values of 31, 32 and 33 cycles. Positive and negative predicting value were 100% and 97,5% respectively, but further work is required to definitively define the clinical performances of the Ebola sGP detection Kit.

## Discussion

Here we described a new diagnosis kit based on mAbs recognizing both sGP and GP for ZEBOV detection in blood or plasma using silver amplification technique combined with immunochromatography. Test interpretation is performed by an easy to use, portable, battery operated reader to standardize and eliminate subjective interpretation, providing clear diagnostic results outside of laboratories and specific areas where patients are likely to be diagnosed. LFA cartridges are ready to use, composed of an immunochromatography assay strip and two small compartments containing a reducing agent and silver ions. The FUJI DRI-CHEM IMMUNO AG1 analyzer (FUJIFILM, Japan) automatically performs the silver amplification step and signal quantification [28]. Results from the stability test showed that the storage of Ebola sGP Detection Kits at 37°C for seventeen weeks does not impact its analytical performance, making this test suitable for use in a high ambient temperature environment (Fig 1D).

Since our goal is to early detect infected patients following onset of symptoms, we choose to target GP proteins (sGP and GP) to maximize the capture and detection of antigens (sGP, shed GP, virions) through the entire course of infection. The main form is a secreted protein encoded by 70–80% of the primary open reading frame of *ebolavirus* GP gene [29]. As expected, significant amounts of sGP were detected in acutely infected humans [8]. Following selection of mAbs and pairing experiments, we identified a pair of mAbs recognizing specifically ZEBOV sGP with a Kd of 10 nM. Selected mAbs also detect the recombinant GP by ELISA, but with a slightly lower intensity. These two mAbs were then used to establish an Ebola sGP Detection Kit.

Here we showed that this Ebola sGP Detection Kit displays a LOD corresponding to 7.99 x 10^5^ genome copies/ml with Vero cells supernatant diluted in blood (Fig 2A), 2.21 x 10^4^ genome copies/ml with plasma samples from a cohort of infected monkeys (Table 2, measured by RT-qPCR). These LODs are in agreement with published results for other RDTs, and even better since none of the immunochromatography-based RDTs tested so far displayed a LOD below 10^5^ genome copies/ml. Furthermore, the human matrix used to dilute plasma samples from infected monkey has no effect on the LOD (Fig 2B and 2C), suggesting that Ebola sGP Detection kit could be used with human blood sample, without loss of sensitivity. In this perspective, the Ebola sGP Detection Kit analytical characteristics should then be evaluated in the future with blood samples collected by finger-stick since only 40 to 70 μl of sample are required. Moreover, following addition of diluted blood or serum in the extraction buffer to the sample pad, no further manipulation is required to get the final result. These criteria are critical to reduce risks of contamination in remote areas and in low income countries, where specialized laboratories are not available.

The delta OD values showed linear relationship with log of genome copies/ml when serial dilution of plasma of infected monkey or cell supernatant were tested (Fig 2B and 2C). As expected, no linear pattern was observed when assessed using values from the different infected monkeys (Table 2) of the cohort underlining the complexity of the multiple host and viral factors involved in the regulation of virus replication, viral particles formation and protein production.

In contrast to other antigenic diagnostic tests targeting EBOV VP40, the Ebola sGP Detection Kit was specific of human EBOV Zaire strain as none of the other hemorrhagic fever viruses tested were positive nor SUDV. This lack of cross-reactivity with SUDV reflects a lower conservation of sGP protein compare to VP40 (S3 Fig). From the first outbreak in 1976 to 2018 excluding the 2013–2016 West Africa major outbreak, percentage of human cases infected with BDBV, SUDV and ZEBOV are 9%, 31% and 60% respectively. As SUDV is responsible for around 1/3 of total cases, we investigated whether some mAbs generated during this work against ZEBOV sGP could cross-react with SUDV sGP as the NH2-terminal part of this protein is conserved across ZEBOV and SUDV (S4 Fig). An additional screen of mAbs was performed against SUDV sGP and identified 3 antibodies strongly reacting against sGP from both ZEBOV and SUDV (XC2, JA7 and GC1, S4 Fig). The ability of these new pairs of antibodies (JA7 & XC2 or GC1 & XC2 or JA7 & GC1) to detect SUDV and ZEBOV by silver amplification coupled with immunochromatography needs to be further investigated.

The sGP Detection Kit test has a sensitivity of 85.7% (18/21), and a specificity of 100% (119/119). This test could then be used as a triage test to reduce nosocomial transmission among patients as it would reduce time spent by negative patients in health care centers [17]. This high specificity is unique to our test as only one out of 4 LFA tests achieves an equivalent specificity in a recent comparative study [30]. Interestingly, all the 8 non-EBOV febrile patients admitted at the Macenta ETC are negative with the sGP Detection Kit as well as all cured-EBOV patients (triangle, Fig 3C). The sensitivity of our test has to be further delineated as only 21 samples were evaluated. It is important to note that among those positive samples, 4 were diagnosed by RT-qPCR with a Ct value ranging from 31 to 33 (Fig 3C). Out of these 4 samples with low viremia, 3 were tested negative with the sGP Detection Kit. This result is in agreement with results obtained with other RDTs using low viremia samples (high Ct, usually above 29 or 30) [30]. Samples from these 4 patients with Ct above 31 are blood draws collected at the recovery phase, i.e. before leaving the Ebola Treatment Center of Macenta. One could therefore argue that anti-ZEBOV antibodies are engaged in immune complex, masking sGP/GP for detection. Anti-ZEBOV IgG titers have been evaluated on viral lysates for these survivors’ patients [31]. All these 4 survivors’ patients are positive for IgG [31]. Those antibodies could be due to prior asymptomatic infection or they have been generated during this infection [32]. Therefore, an antigenic detection kit, whatever the targeted antigen, may never be reliable to follow patient viremia during treatment. However, our goal was to establish an antigenic detection kit to diagnose early infection by mainly targeting sGP (and GP to a lesser extent), which is present at high concentration in blood of infected patients [3, 8]. In these settings, we anticipate that most of the time no anti-ZEBOV antibody responses would be detected. Recent works nevertheless highlighted the detection of anti-ZEBOV antibodies in healthy people, presumably reflecting asymptomatic infections or paucisymptomatic cases, with different seroprevalences [32–34]. Since a long and persistent antibody response is observed in Ebola survivors, we could assume that survivors are protected against re-infection and therefore have minor chance to get infected and spread infection in outbreak settings [35]. Therefore, the presence of specific antibodies for ZEBOV proteins should not interfere when assessing Ebola disease status with antigenic RDTs in naive patients.

World Health Organization has approved seven *in vitro* diagnosis (IVD) tests for emergency use assessment. Among those, five are based on molecular detection of Ebola virus nucleic acids and two are based on Ebola virus VP40 antigen detection: OraQuick Ebola Rapid Antigen Test Kit and Antigen Rapid Test Kit ReEBOV [36, 37]. Here, the described detection limit of the Ebola sGP Detection Kit was almost 10 times lower than WHO approved IVD tests based on antigen detection or recently described RDTs [36, 37]. Since all tests are LFA, identical technical advantages apply to all of them: minimum sample processing and short time to results. In contrast to other LFA with user-dependent reading of the test result, the Ebola sGP Detection Kit relies on portable battery-operated reader, which can help provide clear and unbiased diagnostic results outside of laboratories.

Future investigations are needed in order to confirm the promising results described in this paper. For example, we need to evaluate the sGP Detection Kit sensitivity and specificity with a larger cohort using human finger-stick whole blood, during an outbreak, to assess the capability of this test to identify ZEBOV-infected patients earlier than VP40-based diagnostic tests. Because the sGP detection kit is specific to Zaire EBOV, we also need to evaluate the ability of the 3 ZEBOV- and SUDV-specific mAbs to recognize other EBOV species in LFA.

In conclusion, the LFA developed here together with the easy-to-use reader equipment gave highly specific and sensitive results that could be implemented in low-resource laboratories to help monitoring and diagnosing ZEBOV in countries where outbreaks could start.

## Supporting information

S1 Fig Alignment of sGP proteins from ZEBOV virus Gabon 2002 used in monkey experiments with Makona 2014 used to characterize mAbs generated in this study. Sequences GenBank accession numbers are as follows: EBOV/H.sap/GIN/14 (KT765131) and EBOV/H.sap/GAB/02 (AGB56831). Alignment was performed using MultiAlign website [38].
(TIF)

S2 Fig Screening strategy to select mAbs specific for sGP, GP or both following mice immunization. The different steps used for mAbs screening are depicted as well as the number of mAbs and pairs selected following each step. EIA: Enzyme Immunoassay; ELISA: Enzyme-linked Immunosorbent Assays. See methods for experimental details.
(TIF)

S3 Fig Alignment of VP40 and sGP proteins from several ZEBOV viruses (Democratic Republic of Congo, Guinea, Sierra Leone or Liberia) and one SUDV virus (Uganda), all responsible of outbreaks. All sequences obtained or utilized in this study are available in GenBank. Accession numbers are as follows: EBOV/H.sap/COD/76 (KC242801); EBOV/H.sap/COD/95 (KR867676); EBOV/H.sap/COD/07 (KC242786); EBOV/H.sap/GIN/14 (KT765131); EBOV/H.sap/LBR/14 (KR075003); EBOV/H.sap/SLE/15 (KT357856); and SUDV/H.sap/UGA/00 (KR063670). Alignment was performed using MultiAlign website [38].
(TIF)

S4 Fig Assessing the cross-reactivity of developed mAbs towards sGP from SUDV. Indirect ELISA performed by coating 100 ng/well of sGP (Mayinga in light green or SUDV in light pink) as described in Methods. Monoclonal antibodies were used at 1 μg/ml (1/1000), 0.5 μg/ml (1/2000), 0.25 μg/ml (1/4000) and 0.125 μg/ml (1/8000). Results are OD_450_ of individual well.
(TIF)
