# Supplementary figures and images for: Characterization and analytical validation of a new antigenic rapid diagnostic test for Ebola virus disease detection
Source: PLoS Negl Trop Dis. 2020 Jan 17;14(1):e0007965. doi: 10.1371/journal.pntd.0007965 (PMC6992227; doi:10.1371/journal.pntd.0007965)

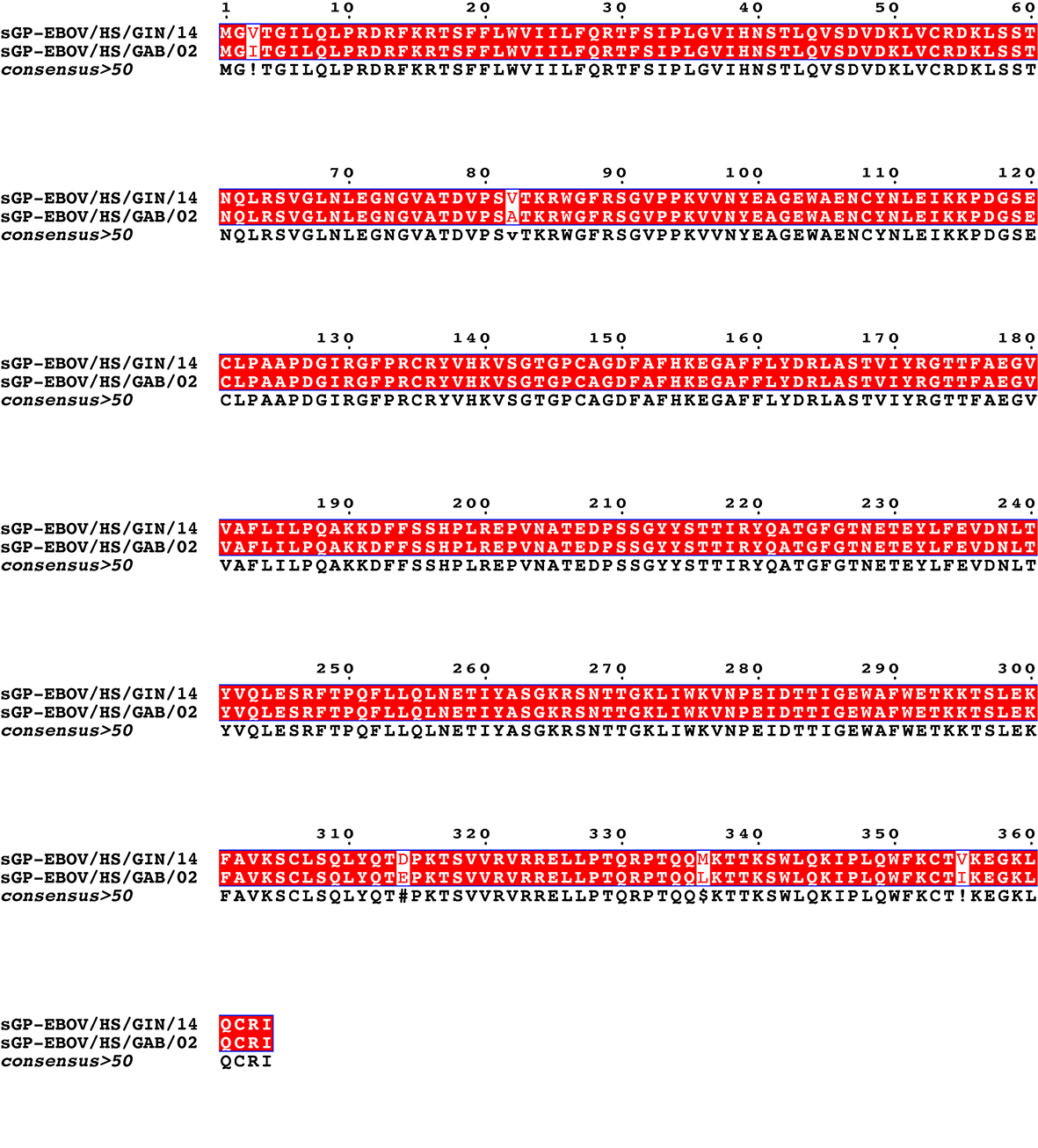

Supplement: S1 Fig — Sequences GenBank accession numbers are as follows: EBOV/H.sap/GIN/14 (KT765131) and EBOV/H.sap/GAB/02 (AGB56831). Alignment was performed using MultiAlign website [38]. (TIF) [file pntd.0007965.s001.tif]

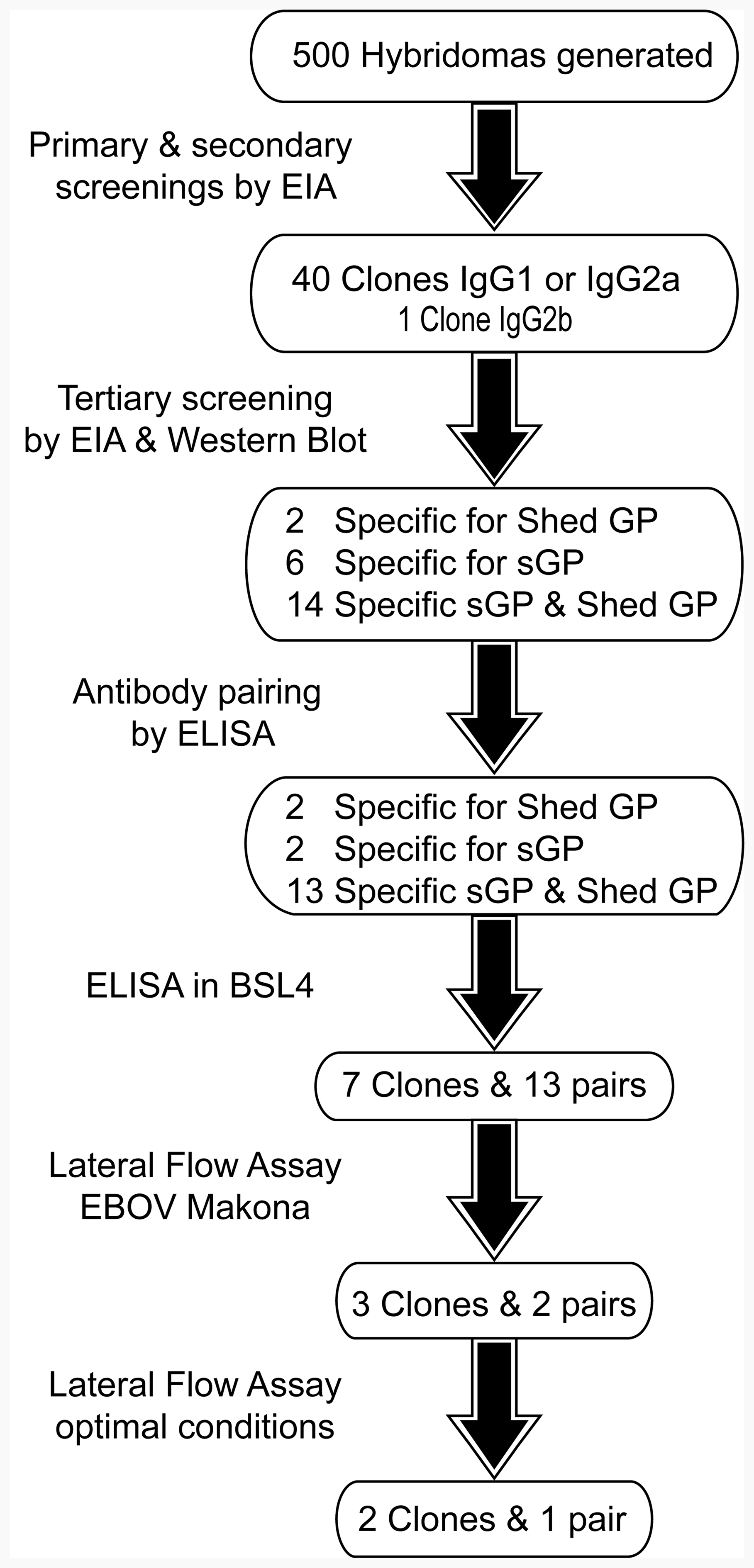

Supplement: S2 Fig — The different steps used for mAbs screening are depicted as well as the number of mAbs and pairs selected following each step. EIA: Enzyme Immunoassay; ELISA: Enzyme-linked Immunosorbent Assays. See methods for experimental details. (TIF) [file pntd.0007965.s002.tif]

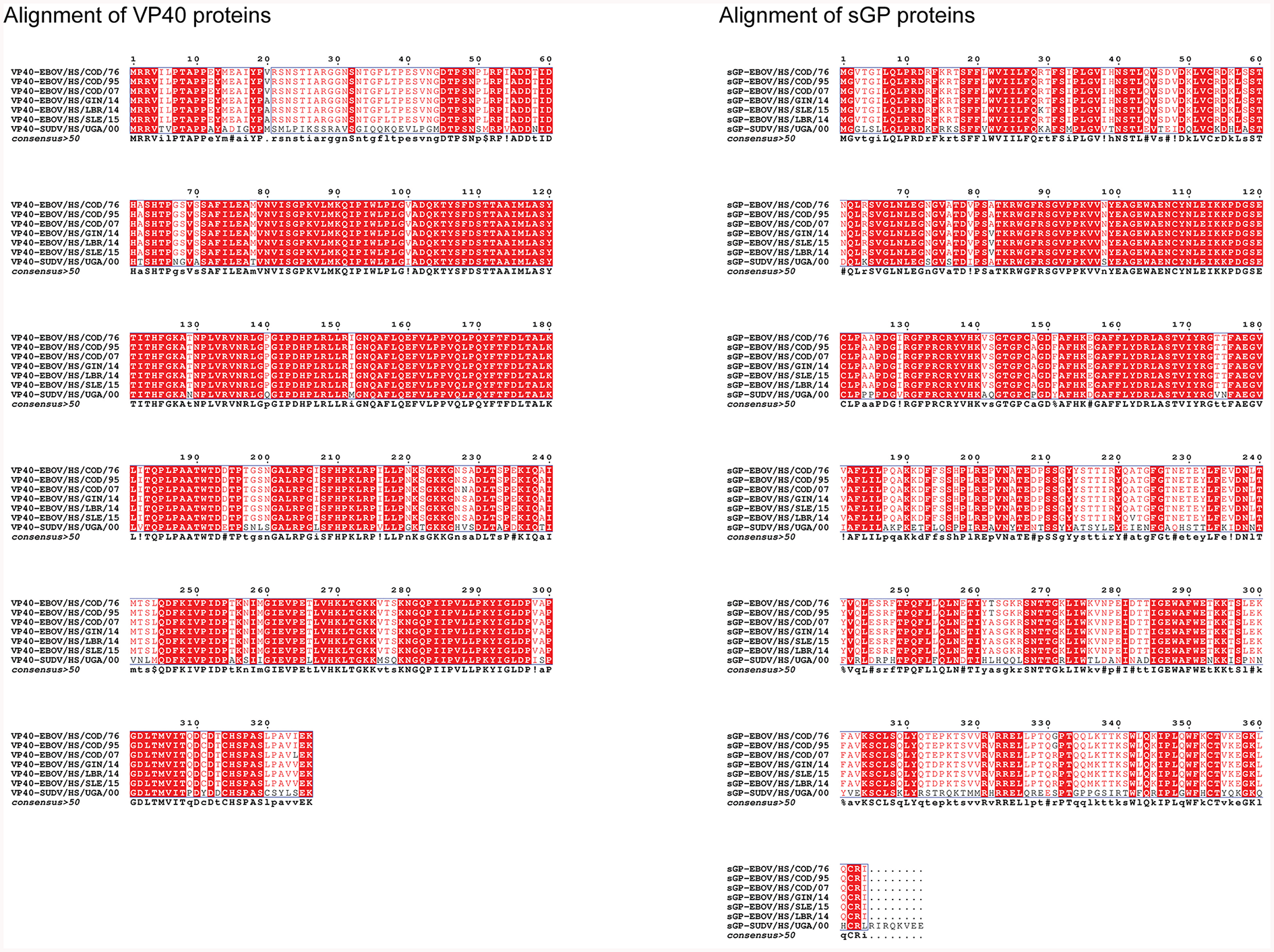

Supplement: S3 Fig — All sequences obtained or utilized in this study are available in GenBank. Accession numbers are as follows: EBOV/H.sap/COD/76 (KC242801); EBOV/H.sap/COD/95 (KR867676); EBOV/H.sap/COD/07 (KC242786); EBOV/H.sap/GIN/14 (KT765131); EBOV/H.sap/LBR/14 (KR075003); EBOV/H.sap/SLE/15 (KT357856); and SUDV/H.sap/UGA/00 (KR063670). Alignment was performed using MultiAlign website [38]. (TIF) [file pntd.0007965.s003.tif]

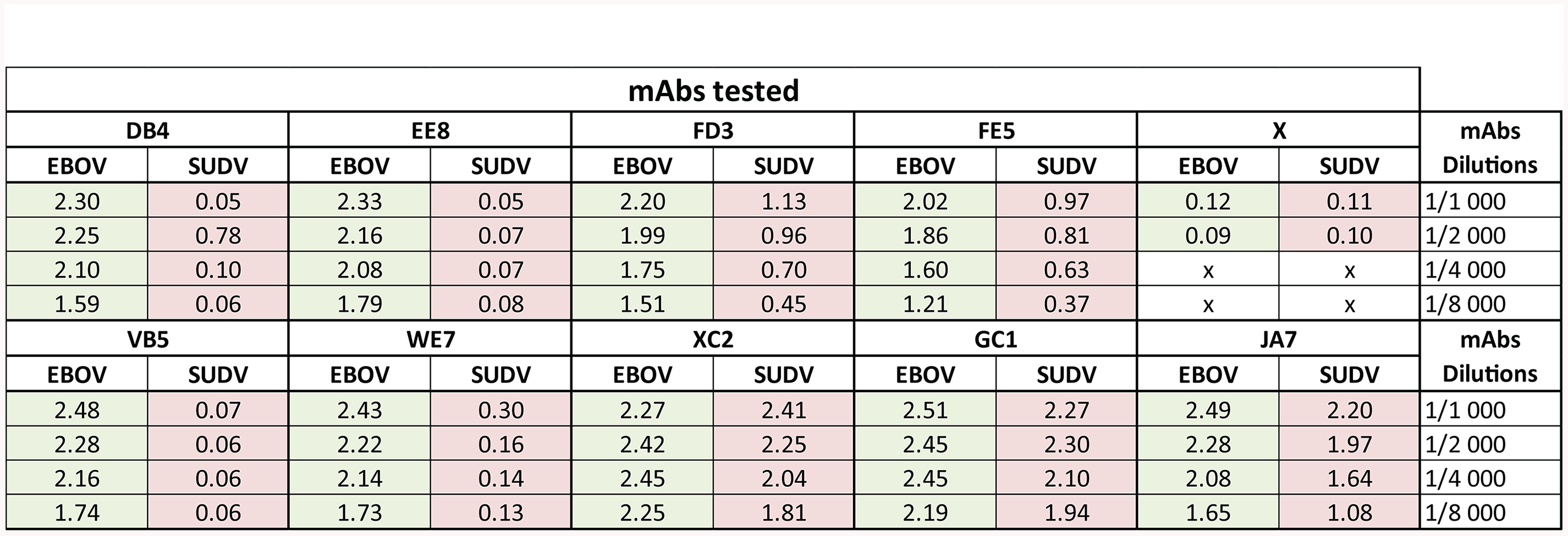

Supplement: S4 Fig — Indirect ELISA performed by coating 100 ng/well of sGP (Mayinga in light green or SUDV in light pink) as described in Methods. Monoclonal antibodies were used at 1 μg/ml (1/1000), 0.5 μg/ml (1/2000), 0.25 μg/ml (1/4000) and 0.125 μg/ml (1/8000). Results are OD450 of individual well. (TIF) [file pntd.0007965.s004.tif]
